# Transcriptome-wide map of N6-methyladenosine (m6A) profiling in coronary artery disease (CAD) with clopidogrel resistance

**DOI:** 10.1186/s13148-023-01602-w

**Published:** 2023-12-15

**Authors:** Ruoyan Yu, Qinglin Yu, Zhenwei Li, Jiyi Li, Jin Yang, Yingchu Hu, Nan Zheng, Xiaojin Li, Yudie Song, Jiahui Li, Xiaomin Chen, Weiping Du, Jia Su

**Affiliations:** 1 Department of Cardiology, The First Affiliated Hospital of Ningbo University, Ningbo, Zhejiang People’s Republic of China; 2 Key Laboratory of Precision Medicine for Atherosclerotic Diseases of Zhejiang Province, Ningbo, People’s Republic of China; 3 Department of Traditional Chinese Internal Medicine, The First Affiliated Hospital of Ningbo University, Ningbo, Zhejiang People’s Republic of China; 4 Department of Cardiology, Yuyao People’s Hospital of Zhejiang Province, Yuyao, Zhejiang People’s Republic of China; 5 Department of Geriatrics, The First Affiliated Hospital of Ningbo University, Ningbo, Zhejiang People’s Republic of China; 6 Department of Cardiology, HwaMei Hospital, University of Chinese Academy of Sciences, Ningbo, Zhejiang People’s Republic of China

**Keywords:** m6A modification, Clopidogrel resistance, Coronary artery disease, RNA transcriptome expression

## Abstract

**Background:**

Clopidogrel resistance profoundly increases the risk of major cardiovascular events in coronary artery disease (CAD) patients. Here, we comprehensively analyse global m6A modification alterations in clopidogrel-resistant (CR) and non-CR patients.

**Methods:**

After RNA isolation, the RNA transcriptome expression (lncRNA, circRNA, and mRNA) was analysed via RNA-seq, and m6A peaks were identified by MeRIP-seq. The altered m6A methylation sites on mRNAs, lncRNAs, and circRNAs were identified, and then, GO and KEGG pathway analyses were performed. Through joint analysis with RNA-seq and MeRIP-seq data, differentially expressed mRNAs harbouring differentially methylated sites were identified. The changes in m6A regulator levels and the abundance of differentially methylated sites were measured by RT-PCR. The identification of m6A-modified RNAs was verified by m6A-IP-qPCR.

**Results:**

The expression of 2919 hypermethylated and 2519 hypomethylated mRNAs, 192 hypermethylated and 391 hypomethylated lncRNAs, and 375 hypermethylated and 546 hypomethylated circRNAs was shown to be altered in CR patients. The m6A peaks related to CR indicated lower mark density at the CDS region. Functional enrichment analysis revealed that inflammatory pathways and insulin signalling pathways might be involved in the pathological processes underlying CR. The expression of mRNAs (ST5, KDM6B, GLB1L2, and LSM14B), lncRNAs (MSTRG.13776.1 and ENST00000627981.1), and circRNAs (hsa_circ_0070675_CBC1, hsa-circRNA13011-5_CBC1, and hsa-circRNA6406-3_CBC1) was upregulated in CR patients, while the expression of mRNAs (RPS16 and CREG1), lncRNAs (MSTRG.9215.1), and circRNAs (hsa_circ_0082972_CBC1) was downregulated in CR patients. Moreover, m6A regulators (FTO, YTHDF3, and WTAP) were also differentially expressed. An additional combined analysis of gene expression and m6A peaks revealed that the expression of mRNAs (such as ST5, LYPD2, and RPS16 mRNAs) was significantly altered in the CR patients.

**Conclusion:**

The expression of m6A regulators, the RNA transcriptome, and the m6A landscape was altered in CR patients. These findings reveal epitranscriptomic regulation in CR patients, which might be novel therapeutic targets in future.

**Supplementary Information:**

The online version contains supplementary material available at 10.1186/s13148-023-01602-w.

## Introduction

Clopidogrel, a novel antiplatelet drug used to treat CAD patients after percutaneous coronary intervention (PCI), reduces platelet aggregation by inhibiting the activity of the adenosine diphosphate (ADP) receptor P2Y_12_. However, approximately 10–30% of treated patients still suffer from ischaemic events [1], which might be due to a reduced degree of platelet inhibition. The inability to effectively prevent platelet aggregation, which results in high residual platelet activity, is considered to be indicative of low response to clopidogrel or clopidogrel resistance. It clearly increases the risk of recurrent cardiovascular and cerebrovascular events in CAD patients after PCI.

Complex factors have been reported to influence the clopidogrel response, including clinical factors (such as diabetes), sequence alterations (SNPs, lncRNAs, miRNAs, and mRNAs), and epigenetic modifications (DNA methylation and RNA methylation). Among these factors, RNA methylation is currently a hot research topic. In most eukaryotes, m6A modification in the 5′ cap region of mRNA plays an important role in the maintenance of mRNA stability, variant splicing, polyadenylation, transportation, and translation, and a m6A mark in the 3′ polyA region contributes to the extracellular transport, initial translation, and structural stability of the mRNA along with polyA-binding proteins [2]. The dynamic modification of m6A methylation is related to life processes such as immune responses and stem cell renewal in the body, which can affect the signalling mediated through downstream molecules, cause gene expression imbalance, and alter cell differentiation, homeostasis, and the stress response, leading to the occurrence of diseases or acquisition of pathological states.

However, few studies have reported the effects of m6A modification on the efficacy of cardiovascular drugs. The previous studies have confirmed that METTL3-mediated m6A modification upregulated the expression of the long-chain intergenic noncoding RNA linc00958, leading to increased adipogenesis and affecting the prognosis of hepatocellular carcinoma chemotherapy [3]. The total saponin content in Panax notoginseng can affect the WTAP/p16 signalling pathway through FTO-mediated m6A modification, thereby inhibiting the proliferation, migration, and intimal hyperplasia of vascular smooth muscle cells [4]. However, whether this dynamic modification can affect the antiplatelet effect of clopidogrel is unknown. In the present study, our group sequenced the whole transcriptome from CAD patients, and then, the m6A methylation abundance of the whole transcriptome was analysed. An integrative analysis of RNAs revealed the molecular mechanism underlying clopidogrel resistance.

## Results

### Characteristics of the patients

There were 46 patients enrolled in our study, and samples from ten patients (five CR and five NC) were used for the systematic analysis of RNA m6A modification abundance, and samples from the remainder of the patients (18 CR and 18 non-CR) were used for validation. The clinical features of the participants are shown in Table 1. Except for that of the platelet function measure, no significant difference in baseline levels was found between the two groups.

**Table 1:** Characteristics of the patients included in the profiling analysis

	CR (*N* = 5)	Non-CR (*N* = 5)	*t*/2	*P* value
PRU	258.80 ± 34.49	164.60 ± 43.14	3.813	0.005
InhibitionPer	0.20 ± 0.12	0.40 ± 0.17	− 2.089	0.070
Baseline	326.80 ± 57.50	275.80 ± 41.81	1.604	0.147
Age (year)	63.40 ± 7.92	59.60 ± 8.84	0.715	0.495
Male, *n* (%)	4 (80)	5 (100)	–	1.000
Hypertension, *n* (%)	4 (80)	3 (60)	–	1.000
Diabetes, *n* (%)	0 (0)	1 (20)	–	1.000
Smoking history, *n* (%)	2 (40)	2 (40)	–	–
Drinking history, *n* (%)	1 (20)	2 (40)	–	1.000
Statin	1.60 ± 0.55	2.40 ± 0.89	− 1.706	0.126
PPI	1.00 ± 1.22	0.60 ± 0.55	0.667	0.524
BMI (kg/m^2^)	23.78 ± 0.87	23.38 ± 2.10	0.393	0.704
Stnet	1.60 ± 0.55	1.00 ± 0.00	2.449	0.070
LVEF (%)	60.60 ± 5.55	61.40 ± 2.51	0.294	0.776
TC (mmol/L)	4.83 ± 1.04	4.80 ± 1.60	0.030	0.977
TG (mmol/L)	1.03 ± 0.50	2.02 ± 0.97	− 2.025	0.077
HDL (mmol/L)	1.29 ± 0.42	0.95 ± 0.19	1.664	0.135
LDL (mmol/L)	2.76 ± 1.16	2.99 ± 1.42	− 0.287	0.781
GLU (mmol/L)	5.94 ± 1.73	5.07 ± 0.51	1.078	0.333
HbA1C (%)	5.60 ± 0.53	5.90 ± 0.12	− 1.225	0.256
ALT (U/L)	31.60 ± 24.58	37.80 ± 32.18	− 0.342	0.741
AST (U/L)	147.20 ± 179.63	89.8 ± 139.98	0.564	0.588
TBIL (µmol/L)	15.16 ± 8.16	14.54 ± 3.14	0.159	0.878
CREA (µmol/L)	72.91 ± 16.15	69.05 ± 6.77	0.493	0.641
UA (µmmol/L)	408.20 ± 262.48	362.80 ± 60.52	0.370	0.721
hsCRP (mg/L)	3.39 ± 4.63	6.93 ± 11.77	− 0.626	0.549
PLT (*109/L)	197.60 ± 53.17	218.80 ± 1.15	− 0.588	0.572
MPV (fL)	7.48 ± 0.72	8.04 ± 1.15	− 0.922	0.384
PCT (ng/ml)	0.15 ± 0.03	0.18 ± 0.04	− 1.134	0.290
PDW (fL)	16.28 ± 0.32	16.38 ± 0.71	− 0.287	0.782

A total of 18 CR and 18 non-CR patients with CAD were analysed for the correlation study. The baseline values of the parameters for these patients relisted in Table 2. In addition to the platelet function index and BMI values, the characteristics of the patients in the validation cohort were closely matched.

**Table 2:** Characteristics of the patients included in the validated cohort

	Total (*n* = 36)	CR (*N* = 18)	Non-CR (*N* = 18)	*T*/2	*P* value
PRU	198.50 ± 74.70	274.44 ± 64.94	149.56 ± 46.90	5.185	0.000
InhibitionPer	0.30 ± 0.20	0.20 ± 0.18	0.41 ± 0.16	− 3.753	0.001
Baseline	280.03 ± 58.64	303.72 ± 46.80	256.33 ± 60.84	2.619	0.013
Age (year)	59.50 ± 9.49	62.00 ± 8.87	57.00 ± 9.67	1.617	0.115
Male, *n* (%)	27 (75.0)	14 (77.8)	13 (72.2)	–	1.000
Hypertension, *n* (%)	24 (66.7)	12 (66.7)	12 (66.7)	–	–
Diabetes, *n* (%)	4 (11.1)	4 (22.2)	0 (0.0)	–	0.104
Hyperlipidaemia, *n* (%)	19 (52.8)	8 (44.4)	11 (61.1)	–	0.505
Smoking history, *n* (%)	13 (36.1)	6 (33.3)	7 (38.9)	–	1.000
Drinking history, *n* (%)	8 (22.2)	3 (16.7)	5 (27.8)	–	0.691
Statin	1.75 ± 0.81	1.61 ± 0.78	1.89 ± 0.83	− 1.035	0.308
PPI	0.83 ± 0.65	0.94 ± 0.73	0.72 ± 0.57	1.019	0.315
BMI (kg/m^2^)	23.96 ± 2.96	24.01 ± 2.36	23.90 ± 3.53	0.118	0.907
Stnet	1.25 ± 0.94	1.61 ± 0.85	0.89 ± 0.90	2.475	0.018
LVEF (%)	58.61 ± 8.71	60.16 ± 9.32	57.06 ± 8.01	1.072	0.291
TC (mmol/L)	4.50 ± 1.28	4.62 ± 1.01	4.37 ± 1.52	0.568	0.574
TG (mmol/L)	1,51 ± 0.90	1.45 ± 0.69	1.57 ± 1.09	− 0.373	0.711
HDL (mmol/L)	1.03 ± 0.30	1.08 ± 0.38	0.98 ± 0.20	1.022	0.314
LDL (mmol/L)	2.64 ± 1.00	2.78 ± 1.06	2.51 ± 0.95	0.799	0.430
GLU (mmol/L)	5.74 ± 1.37	5.49 ± 1.36	6.00 ± 1.36	− 1.111	0.274
HbA1C (%)	5.99 ± 0.79	5.99 ± 0.93	5.98 ± 0.65	0.021	0.984
ALT (U/L)	39.67 ± 36.23	38.94 ± 31.15	40.39 ± 41.60	− 0.118	0.907
AST (U/L)	100.42 ± 178.88	111.56 ± 172.84	89.28 ± 189.06	0.369	0.714
TBIL (µmol/L)	15.29 ± 7.13	14.08 ± 8.06	16.50 ± 6.05	− 1.020	0.315
Albumin (g/L)	39.46 ± 3.36	39.36 ± 3.43	39.56 ± 3.38	− 0.171	0.865
BUN (mmol/L)	5.17 ± 1.04	5.20 ± 1.23	5.15 ± 0.84	0.148	0.884
CREA (µmol/L)	72.88 ± 17.85	71.91 ± 16.80	73.86 ± 19.29	− 0.324	0.748
UA (µmmol/L)	343.42 ± 120.03	325.33 ± 145.85	361.50 ± 87.74	− 0.901	0.374
hsCRP (mg/L)	6.89 ± 10.20	5.49 ± 10.51	8.30 ± 9.98	− 0.824	0.416
PLT (*109/L)	208.06 ± 80.34	197.17 ± 62.30	218.94 ± 95.69	− 0.809	0.424

### RNA transcriptome expression profiles in CR and non-CR patients

To further explore the relationship between gene expression and m6A modification, we measured the levels of potential markers of heterogeneity after clopidogrel treatment. We characterized the transcriptome in CR and non-CR patients by RNA-seq with high throughput on the basis of circRNAs, lncRNAs, and mRNAs. With thresholds set to |log2FC|> 1 and a *P* value < 0.05, we identified 583 lncRNAs (391 downregulated and 192 upregulated) and 922 mRNAs (546 downregulated and 376 upregulated). In the light of the expression profile of the circRNA microarray, long probes and short probes were used, and 5448 dysregulated circRNAs (2529 downregulated and 2919 upregulated) were identified at threshold levels of an |log_2_FC|> **1** and a *P* value < 0.05 (Additional file 1: Stable 1, Additional file 2: Stable 2, and Additional file 3: Stable 3).

### Overall characteristics of the m6A modification in CR and non-CR patients

**Fig. 1:**
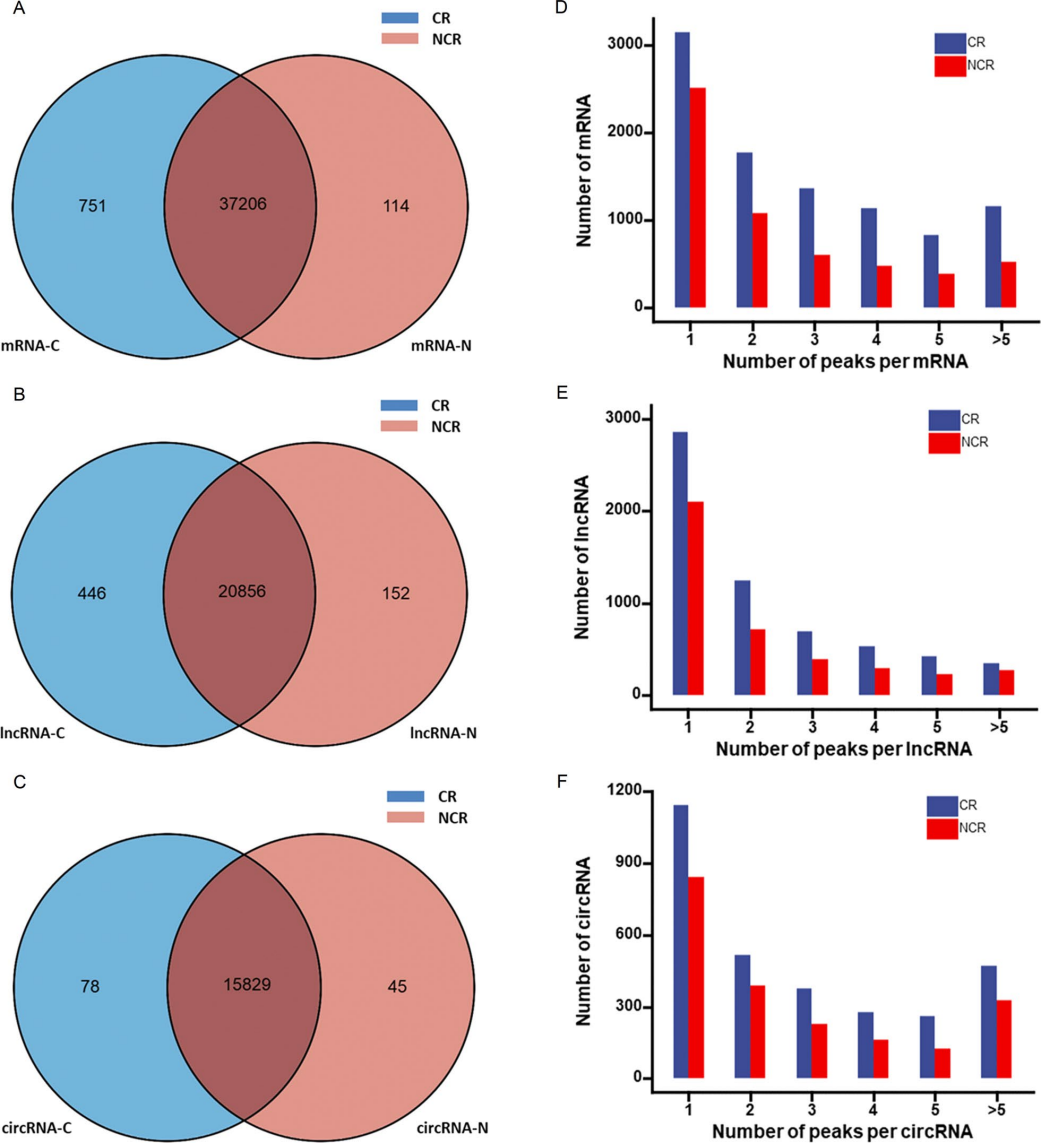
Overview of m6A modification peaks on mRNAs, lncRNAs, and circRNAs in CR and non-CR patients. **A**–**C** Venn diagram depicting the common and distinct m6A peaks in mRNAs, lncRNAs, and circRNAs between CR and non-CR patients. **D**–**F** The numbers of m6A peaks located on mRNA, lncRNA, and circRNA between CR and non-CR patients

Blood samples from ten patients (five CR and five non-CR) were collected and used for MeRIP-seq analysis. As the Venn diagram in Fig. 1A–C displays, 38,071 m^6^A peaks on mRNAs, 21,454 m^6^A peaks of lncRNAs, and 15,944 m^6^A peaks on circRNAs were discovered between CR and non-CR patients. Among these peaks, 751 nonoverlapping m6A peaks were found on mRNAs (Fig. 1A), 446 nonoverlapping m6A peaks were found on lncRNAs (Fig. 1B), and 78 nonoverlapping m6A peaks were found on circRNAs (Fig. 1C) in CR patients. In addition, 114 nonoverlapping m^6^A peaks were found on mRNAs (Fig. 1A), 152 nonoverlapping m^6^A peaks were found on lncRNAs (Fig. 1B), and 45 nonoverlapping m^6^A peaks were found on circRNAs (Fig. 1C) in the non-CR patients.

Then, we measured the distribution of m^6^A marks in the whole transcriptome. The vast majority of m6A-modified RNAs were covered by fewer than 5 m^6^A peaks, and a small number were covered by more than 5 peaks (Fig. 1E–F).

**Fig. 2:**
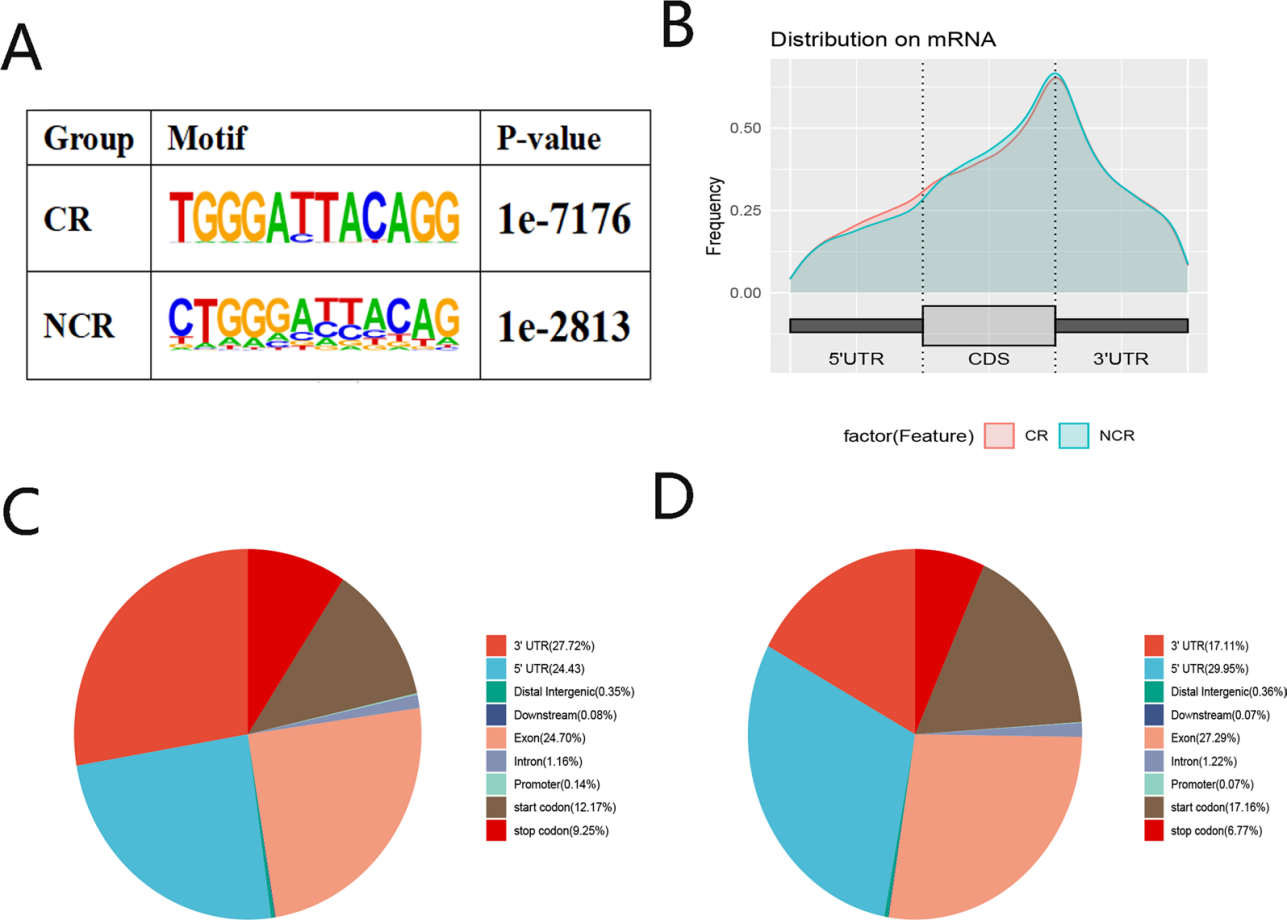
Overview of m6A peaks located on mRNAs, lncRNAs, and circRNAs in CR and non-CR patients. **A** Motifs enriched of all the identified m6A peaks in CR and non-CR patients. **B** Diversity of the m6A peak density in the indicated regions between CR and non-CR patients. **C** Pie chart revealing the regional distributions of m6A peaks in the RNA transcriptome of CR patients. **D** Pie chart revealing the regional distributions of m6A peaks in the RNA transcriptome of non-CR patients

We used HOMER motif software to analyse the m^6^A peaks. In contrast with that in the CR groups, the motif sequence in the mRNAs was GGACC in the non-CR groups (Fig. 2A), which was consistent with the previously obtained results. To explore the potential function of m6A, we measured the distribution of m6A peaks across the whole transcriptome. We found that the m6A peaks were mostly enriched at the start of the 3′UTR in both the CR and non-CR patients samples (Fig. 2B). m6A peaks in the CR patient samples showed a lower density in the CDS region than those in the non-CR patient samples. In addition, the distribution of m6A peaks in the CR and non-CR patient samples showed had the same tendency, increasing from the 5′UTR to the start of the 3′UTR and decreasing from the start of the 3′UTR to the end of the transcriptome. We determined that the proportion of m6A peaks was the largest in the 5′UTR and the smallest in the intron in the non-CR patient sample (Fig. 2C and D).

### Differential m6A-modified RNAs in CR patients samples

**Fig. 3:**
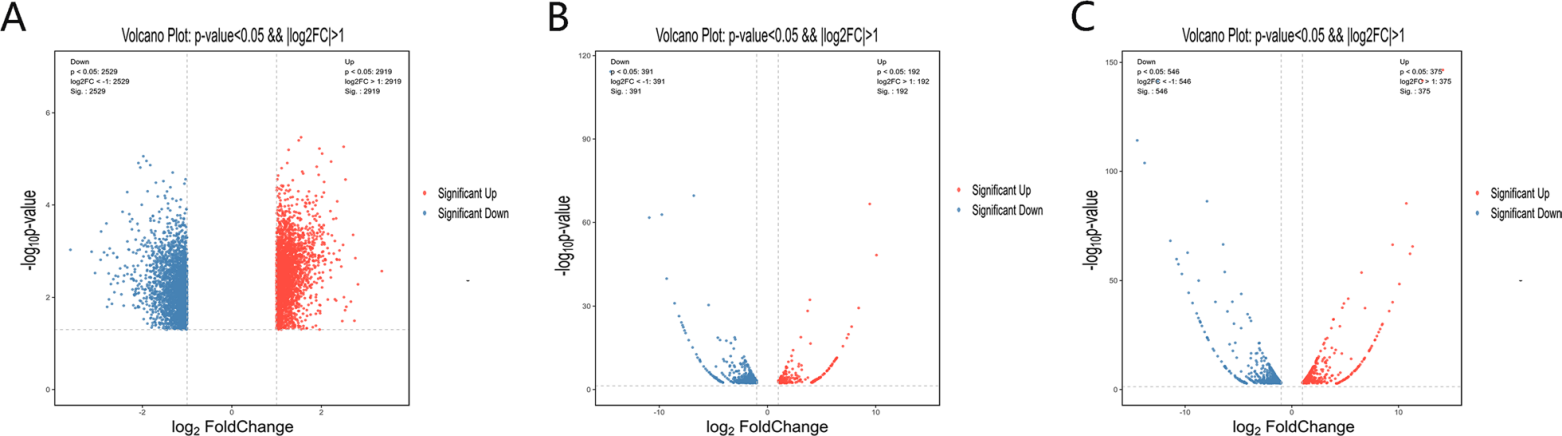
Abnormally m6A-modified mRNAs, lncRNAs, and circRNAs were identified in CR patient samples compared to those in non-CR patients samples. **A** mRNAs, **B** lncRNAs, and **C** circRNAs

**Fig. 4:**
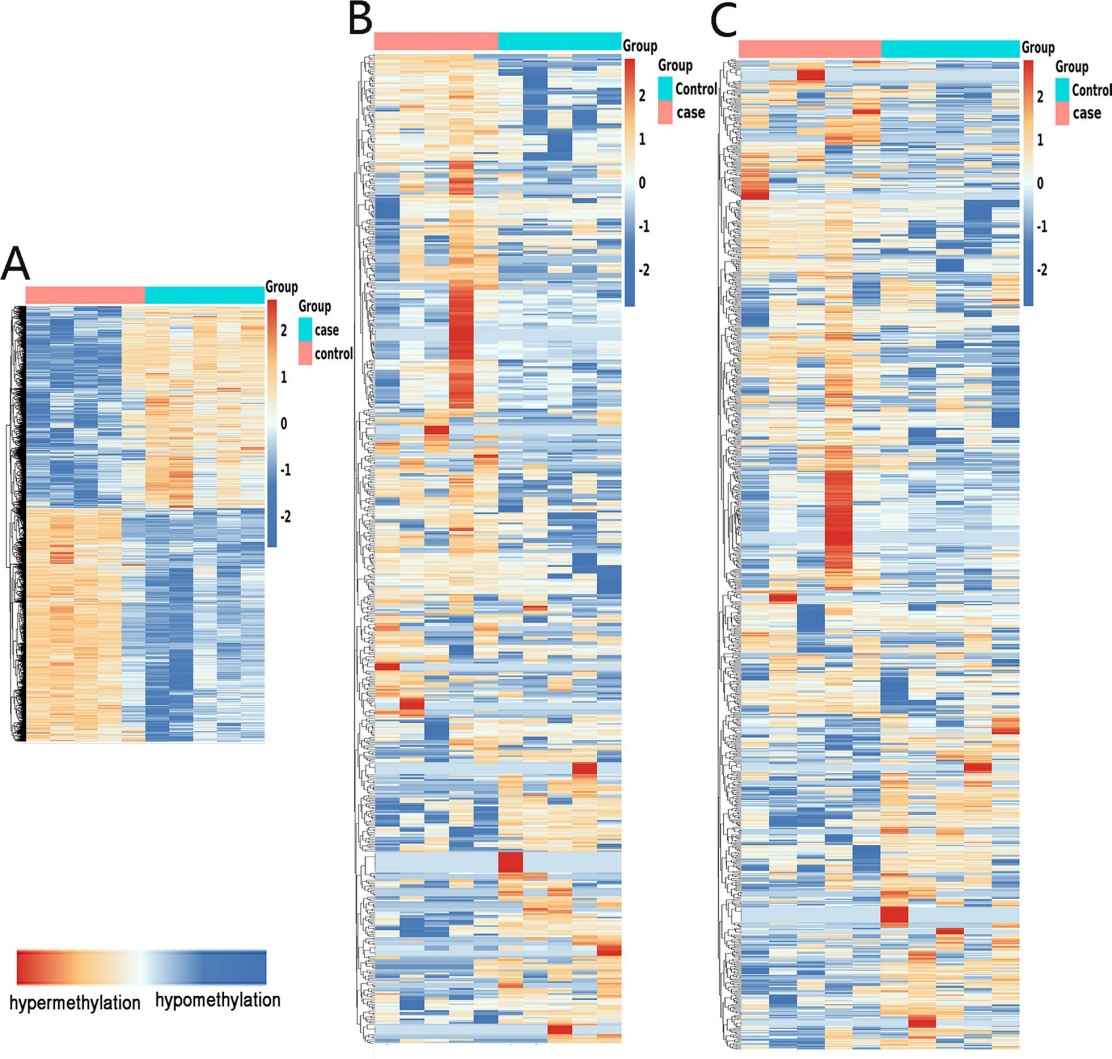
Hierarchical clustering analysis showing the differences in the RNA transcriptome in CR and non-CR patients based on |log_2_FC|> 1 and *P* value < 0.05 criteria. **A** circRNAs, **B** lncRNAs, and **C** mRNAs. In heatmaps, red indicates hypermethylation, and green indicates hypomethylation

Differential m6A-modified mRNAs, lncRNAs, and circRNAs in CR patients samples were analysed and compared to those in the non-CR patients samples. Based on an absolute value of log_2_FC greater than 1 and a *P* value less than 0.05, 2919 hypermethylated and 2519 hypomethylated mRNAs were identified, as shown in volcano plots (Fig. 3A). In addition, 192 hypermethylated and 391 hypomethylated lncRNAs (Fig. 3B) and 375 hypermethylated and 546 hypomethylated circRNAs (Fig. 3C) were identified. According to a heatmap (Fig. 4A–C), we found apparent differences in m6A modification peaks among the three kinds of RNAs between CR and non-CR patient samples in a hierarchical clustering analysis. The ten most hypermethylated and most hypomethylated m^6^A peaks on mRNAs, lncRNAs, and circRNAs of CR patient samples are listed in Tables 3 and 4.

**Table 3:** The ten most hypermethylated m6A modification peaks in CR and non-CR patients

Chrom	txStart	txEnd	GeneName	Fold change	*P* value
*mRNA*					
chr19	3,630,381	3,630,600	PIP5K1C	2.466541353	1.85476E−11
chr14	93,673,174	93,673,459	C14orf142	2.199620973	1.01067E−10
chr19	10,334,481	10,335,020	S1PR2	3.236576121	1.0939E−10
chr16	57,546,661	57,546,782	CCDC102A	5.4175	3.09279E−10
chr8	8,233,762	8,234,280	SGK223	2.391506444	8.10486E−10
chr6	29,640,761	29,640,960	ZFP57	147	1.08661E−09
chr21	46,396,594	46,396,660	FAM207A	3.407960199	1.45722E−09
chr16	3,534,941	3,535,260	NAA60	2.006998346	1.44972E−09
chr7	77,378,740	77,378,920	RSBN1L	3.446861405	1.56042E−09
chr19	49,813,296	49,813,423	SLC6A16	2.193574959	1.68245E−09
*lncRNA*					
chr6	29,705,821	29,706,180	HLA-F-AS1	5.401433692	1.81634E−09
chr6	170,581,681	170,581,880	FLJ38122	10.92079208	3.6416E−09
chr22	28,396,961	28,397,200	TTC28-AS1	6.412244898	8.50001E−09
chr16	3,174,487	3,174,608	RP11-473M20.14	8.161616162	1.37529E−08
chr19	10,574,468	10,574,660	PDE4A	3.523758099	1.57046E−08
chr8	145,676,661	145,677,120	CYHR1	2.912176021	1.60788E−08
chr13	99,739,321	99,739,620	DOCK9-AS2	3.486910995	1.65164E−08
chr22	40,982,781	40,982,980	RPL4P6	6.583850932	2.08606E−08
chr17	80,212,181	80,212,380	CSNK1D	9.931506849	2.38251E−08
chr7	76,256,441	76,256,660	LOC100133091	6.496969697	2.39618E−08
*circRNA*					
chr11	77,458,080	77,458,173	RSF1	2.478178368	8.05427E−06
chr11	17,332,371	17,332,557	NUCB2	2.39638865	6.07573E−06
chr9	74,360,181	74,360,495	TMEM2	2.040329617	5.49828E−06
chr9	710,803	710,880	KANK1	5.570093458	3.28767E−06
chr15	29,346,501	29,347,038	APBA2	2.013734114	2.69503E−06
chr4	106,157,201	106,157,480	TET2-AS1	2.497607656	2.19468E−06
chr6	42,750,421	42,750,680	GLTSCR1L	2.551066218	2.14826E−06
chr13	52,971,561	52,971,760	THSD1	5.216494845	2.1331E−06
chr19	50,222,713	50,222,880	G041290	2.594374236	1.7083E−06
chr6	7,414,501	7,414,623	RIOK1	2.760497667	1.44224E−06

**Table 4:** The ten most hypomethylated m6A modification peaks in CR and non-CR patients

Chrom	txStart	txEnd	GeneName	Fold change	*P* value
*mRNA*					
chr20	60,962,120	60,962,192	RPS21	2.993740973	2.64999E−12
chr20	35,293,638	35,293,704	NDRG3	2.242256637	1.31838E−11
chr7	100,472,700	100,472,780	SRRT	4.387820513	8.51562E−11
chr9	19,380,121	19,380,204	RPS6	2.292633063	9.61683E−11
chr15	57,929,892	57,929,972	MYZAP	8.713178295	9.02203E−11
chr15	83,532,918	83,533,011	HOMER2	5.417475728	1.33388E−10
chr20	33,519,741	33,519,780	GSS	2.913621262	5.04332E−10
chr19	47,700,489	47,700,600	SAE1	2.61566411	4.92249E−10
chr17	72,862,252	72,862,366	FDXR	5.519906323	5.56815E−10
chr17	55,927,199	55,927,417	MRPS23	2.462739993	6.29816E−10
*lncRNA*					
chr19	7,971,001	7,971,280	MAP2K7	4.475232198	2.13882E−10
chr8	42,697,701	42,697,931	THAP1	59.4	3.96023E−10
chr20	2,473,381	2,473,676	ZNF343	17.50724638	2.07725E−09
chr19	18,283,641	18,283,687	PIK3R2	32.7027027	2.61783E−09
chr9	139,750,501	139,750,671	MAMDC4	20.65384615	3.42769E−09
chr15	40,830,561	40,830,800	C15orf57	53.8	4.65413E−09
chr8	29,885,981	29,886,416	MAP2K1P1	5.842883549	5.01133E−09
chr7	55,800,041	55,800,124	SUMO2P3	12.31034483	8.45214E−09
chr17	28,959,441	28,959,640	LRRC37BP1	9.2421875	1.32526E−08
chr20	44,526,061	44,526,280	CTSA	2.054598954	1.65197E−08
*circRNA*					
chr20	35,293,638	35,293,704	NDRG3	2.242256637	1.31838E−11
chr15	83,532,918	83,533,011	HOMER2	5.417475728	1.33388E−10
chr15	43,773,092	43,773,220	TP53BP1	3.273049645	1.15169E−09
chr20	2,473,381	2,473,471	ZNF343	17.50724638	2.07725E−09
chr8	54,923,041	54,923,052	TCEA1	13.69230769	1.36448E−08
chr9	123,249,571	123,249,680	CDK5RAP2	6.1	2.22495E−08
chr7	6,618,131	6,618,200	ZDHHC4	8.345971564	2.41726E−08
chr20	3,970,201	3,970,480	RNF24	3.69119421	9.76761E−08
chr8	26,248,821	26,249,200	BNIP3L	2.235542705	2.09841E−07
chr11	70,269,045	70,269,101	CTTN	2.654643253	2.93602E−07

**Fig. 5:**
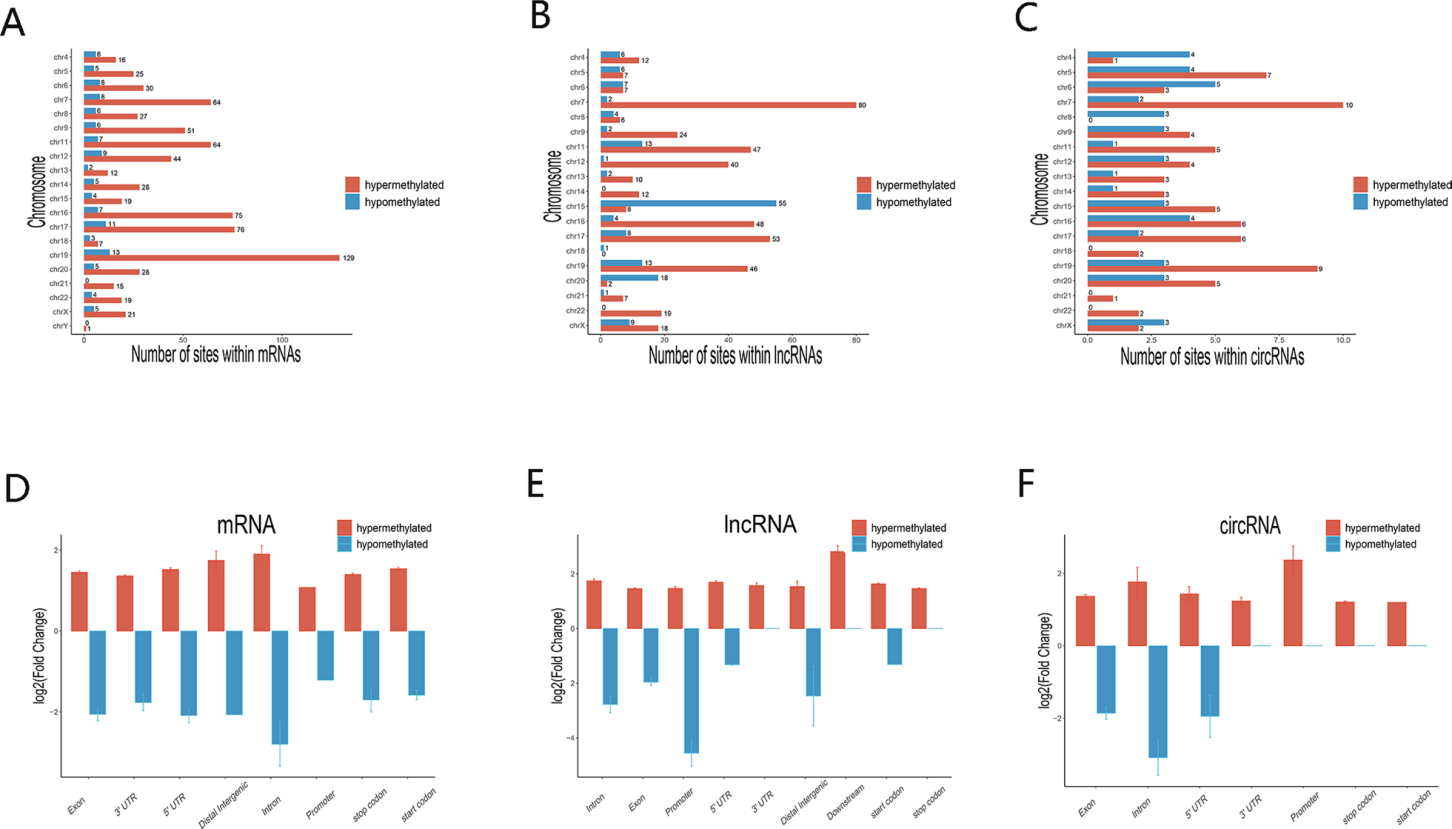
Differences in the chromosomal distribution of differentially methylated m6A sites between CR and non-CR patients. **A**–**C** Chromosomal distribution of differentially methylated m6A sites in three different kinds of RNAs. **D**–**F** Statistical analysis showed fold change differences in hypermethylated and hypomethylated m6A sites in transcript regions of mRNA, lncRNA, and circRNA in CR versus non-CR patients

To measure the distribution of differentially methylated mRNAs, lncRNAs, and circRNAs in chromosomes, we classified differentially hypermethylated and hypomethylated m6A sites by chromosome. We found that the three chromosomes containing the most hypermethylated and hypomethylated m6A sites in mRNAs, lncRNAs, and circRNAs were chromosomes 19 (213), 7 (166), and 17 (152), which are shown in Fig. 5A–C. For mRNAs, the most hypermethylated m6A sites were located on chromosomes 19(129), 17(76), and 16(75); while the most hypomethylated m6A sites were located on chromosomes 19(13), 17(11), and 12(9), as shown in Fig. 5A. For lncRNAs, the most hypermethylated m6A sites were located on chromosomes 7(80), 17(53), and 6(48); while the most hypomethylated m6A sites were located on chromosomes 15(55), 20(18), and 11(13), as shown in Fig. 5B. For circRNAs, the most hypermethylated m6A sites were located on chromosomes 7(10), 19(9), and 5(7); while the most hypomethylated m6A sites were located on chromosomes 6(5), 4(4), and 5(4), as shown in Fig. 5C.

These hypermethylated and hypomethylated m6A sites in mRNA, lncRNA, and circRNA were then classified by transcript region. As shown in Fig. 5D, the fold change was highest for introns with both hypermethylated and hypomethylated m6A sites in mRNA. For the hypermethylated sites in lncRNAs, the fold change was the highest downstream. For the hypomethylated sites in lncRNAs, the fold change was the highest in the promoter (Fig. 5E). Moreover, the fold change was the highest in the promoter for hypermethylated sites in circRNA, and then, it was the highest in the intron for hypomethylated sites in circRNA (Fig. 5F). From all these results, we concluded that there were distinct differences in m6A marks both in terms of their distribution and in their abundance between CR and non-CR patients, indicating that different m6A modification patterns may lead to clopidogrel resistance.

### Functional annotation of altered m6A-modified mRNAs, lncRNAs, and circRNAs between CR and non-CR patients

To characterize the potential function of differential m6A methylation patterns between CR and non-CR patients, mRNAs, lncRNAs, and circRNAs with distinct m6A abundance levels were subjected to Gene Ontology (GO) enrichment analysis and Kyoto Encyclopedia of Gene and Genomes pathway analysis.

**Fig. 6:**
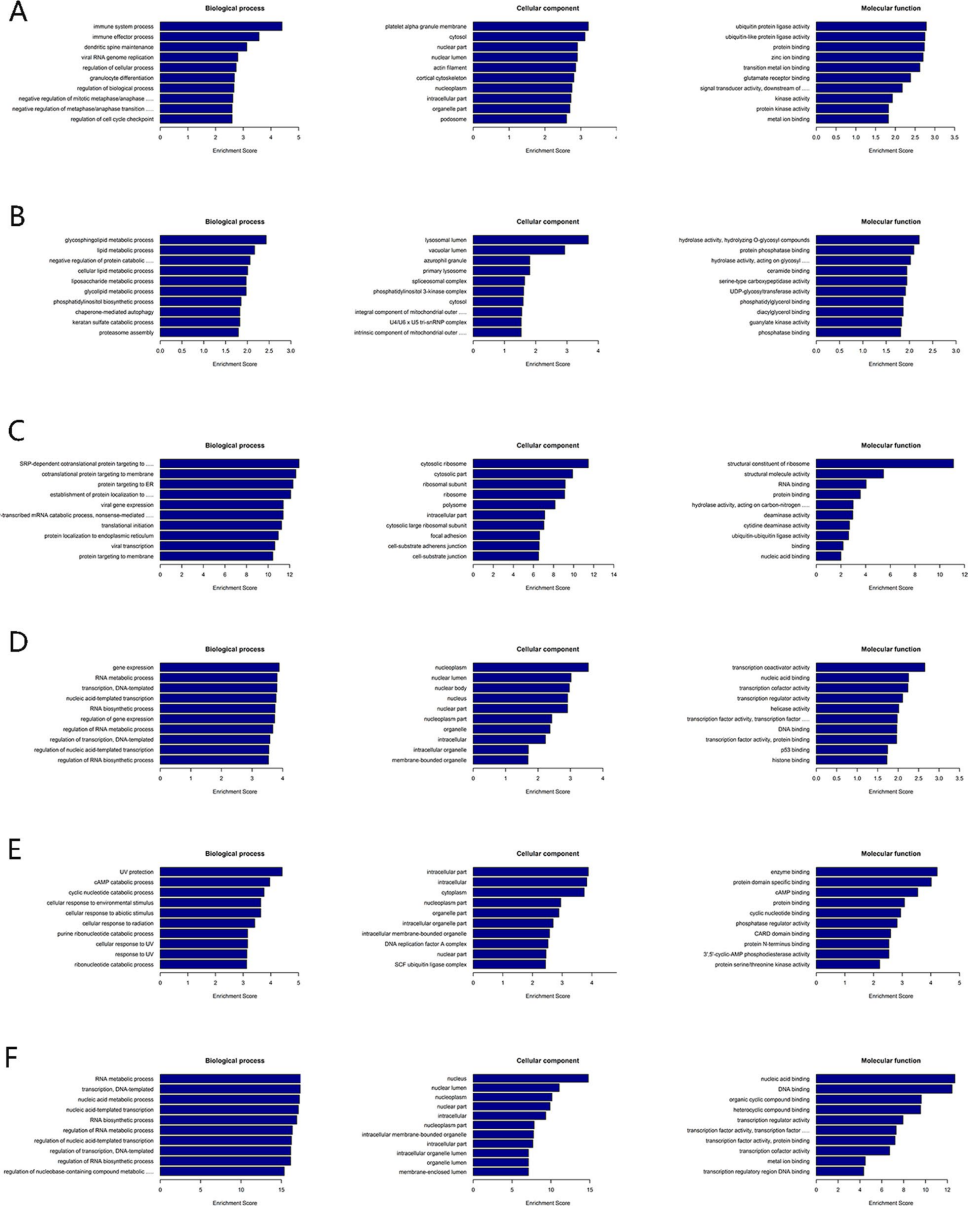
Gene Ontology enrichment analysis of differentially abundant m6A marks between CR and non-CR patients. **A** circRNAs in CR patients, **B** lncRNAs in CR patients, **C** mRNAs in CR patients, **D** circRNAs in non-CR patients, **E** lncRNAs in non-CR patients, and **F** mRNAs in non-CR patients

**Fig. 7:**
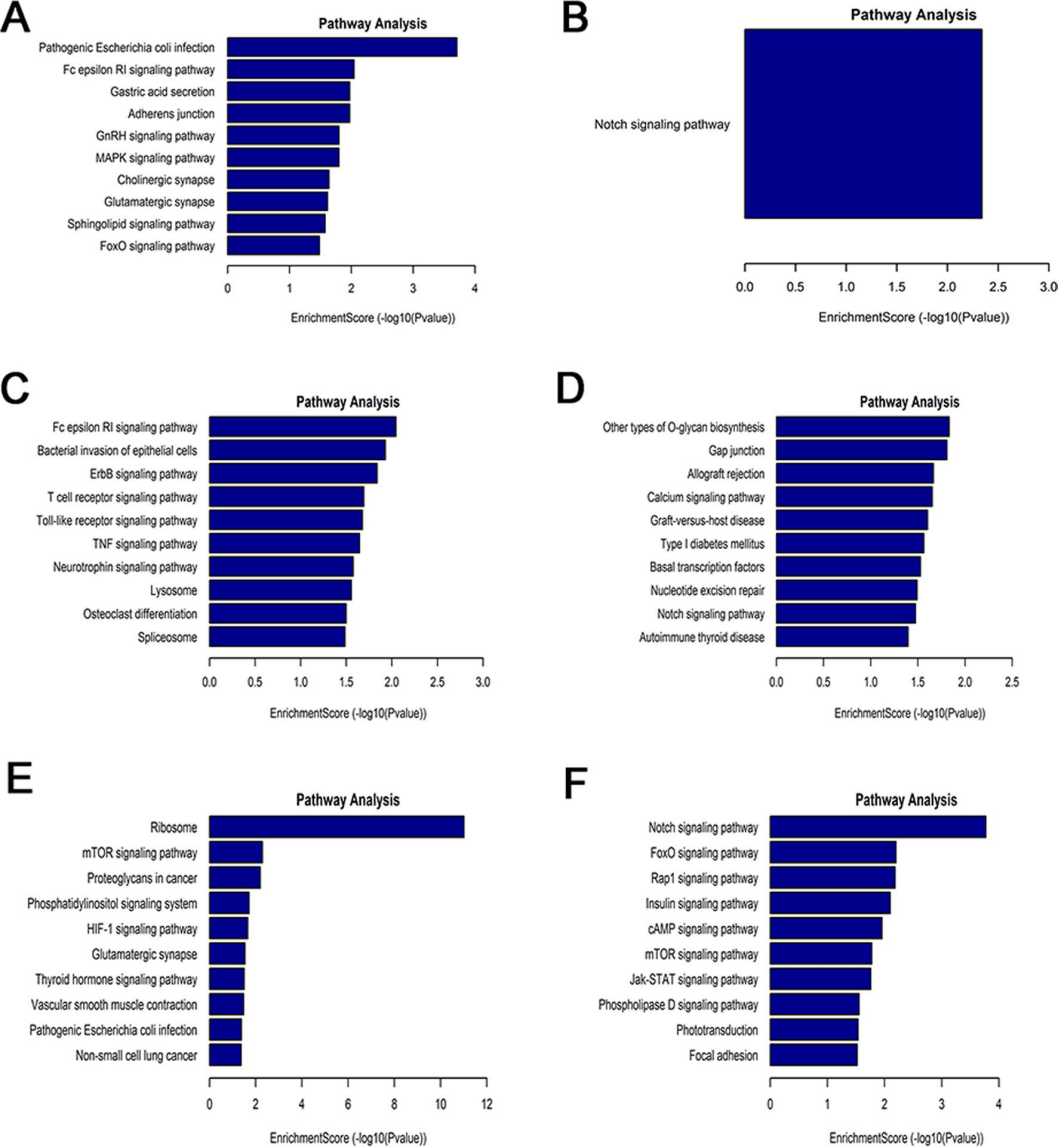
KEGG pathway analysis results showing differentially abundant m6A marks between CR and non-CR patients. **A** circRNAs in CR patients, **B** circRNAs in non-CR patients, **C** lncRNAs in CR patients, **D** lncRNAs in non-CR patients, **E** mRNAs in CR patients, and **F** mRNAs in non-CR patients

GO and KEGG enrichment analyses with circRNA-associated genes that were differentially methylated were performed. For the biological process category, altered m6A-modified genes were significantly interrelated with the regulation of the RNA biosynthetic process (Fig. 6A and D). For the cell composition category, hypomethylated circRNA-associated genes were mainly enriched in platelet alpha granule membrane, cytosol, and nuclear part (Fig. 6A), while hypermethylated circRNA-associated genes were significantly associated with the nucleoplasm, nuclear lumen, and nuclear body (Fig. 6D). For the molecular function category, downregulated circRNA-associated genes were involved in ubiquitin-like protein ligase activity, ubiquitin protein ligase activity, and protein binding (Fig. 6A), while upregulated circRNA-associated genes were mainly enriched in nucleic acid binding, transcription coactivator activity, and transcription cofactor activity (Fig. 6D). KEGG pathway analysis indicated that circRNA-associated genes were enriched in inflammatory pathways (such as the MAPK, Wnt, and Notch signalling pathways) (Fig. 7A and B).

In Fig. 6B, for the BP, CC, and MF categories, the hypomethylated lncRNA-associated genes were enriched mainly in lipid metabolic process, glycosphingolipid metabolic process, and negative regulation of protein catabolic process; lysosomal lumen, vacuolar lumen, and primary lysosome; hydrolyzing O-glycosyl compounds, protein phosphatase binding, and acting on glycosyl bonds, respectively. The hypermethylated lncRNA-associated genes, as displayed in Fig. 6E, were enriched mainly in UV protection, cAMP catabolic process, and cyclic nucleotide catabolic process (BP); intracellular part, intracellular, and cytoplasm (CC); enzyme binding, protein domain-specific binding, and cAMP binding (MF). KEGG pathway analysis revealed that differentially methylated lncRNA-associated genes were also significantly related to inflammatory pathways (such as Notch and Toll-like receptor) (Fig. 7C and D).

In Fig. 6C, for the biological process category, mRNAs harboured m6A sites with downregulated methylation were involved in cotranslational protein targeting to the membrane, SRP-dependent cotranslational protein targeting to the membrane, and protein targeting to the ER. However, m6A sites with upregulated methylation were highly involved in nucleic acid metabolic processes, transcription, DNA templating, and RNA metabolic processes (Fig. 6F). For CC terms, downregulated m6A site methylation was significantly enriched in cytosolic ribosome, cytosolic part, and ribosomal subunit (Fig. 6C), while upregulated m6A site methylation was enriched in the nucleus, nuclear lumen, and nucleoplasm (Fig. 6F). Finally, these mRNAs were significantly correlated with nucleic acid binding, an MF category term (Fig. 6C and F). Furthermore, these downregulated mRNAs were found to be significantly involved in the insulin signalling pathway, VEGF signalling pathway, and so on. The upregulated mRNAs were also involved in the insulin signalling pathway and some inflammatory pathways (such as Notch and mTOR) (Fig. 7E and F).

### Comprehensive analysis of MeRIP-seq and mRNA-seq data in CR and non-CR patients

**Fig. 8:**
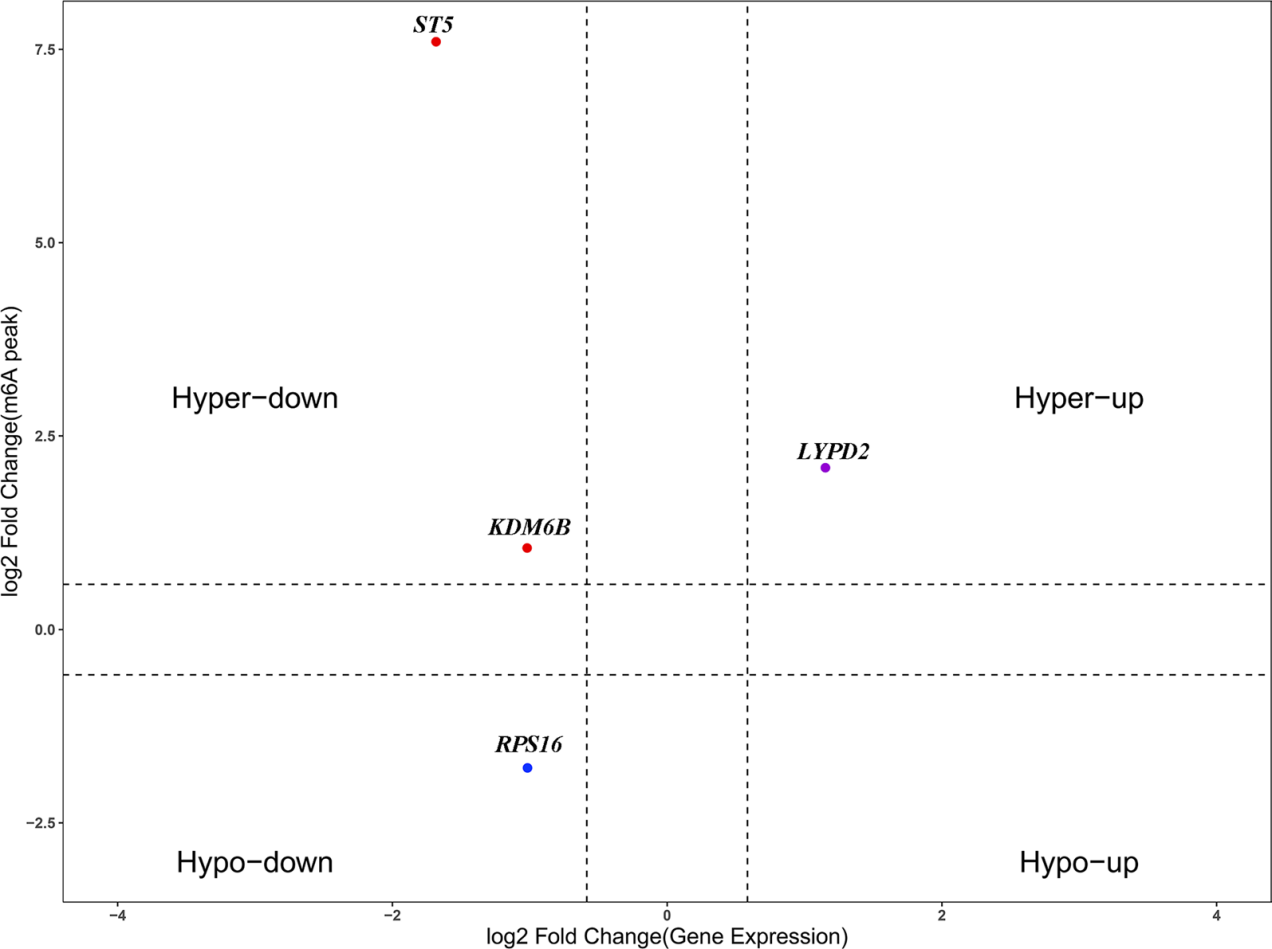
Comprehensive analysis of mRNA m6A peaks and their expression between CR and non-CR patients. Scatter plot showing the distribution of mRNAs with both significantly changed m6A and mRNA levels in CR and non-CR patients

The 25 most highly differentially expressed mRNAs are displayed in Table 5. Through a comprehensive analysis of the data from RNA-seq and MeRIP-seq of CR and NCR patients, we identified one hypermethylated and upregulated (hyper-up) gene (LYPD2), one hypomethylated and downregulated (hypo-down) gene (RPS16), and two hypermethylated and downregulated (hyper-down) genes (KDM6B and ST5) with differential expression in CR patients compared to that in non-CR patients (Fig. 8).

**Table 5:** The 25 most differentially expressed mRNAs based on *P* value

GeneID	logFC	*P* value	FDR	Up/down
TMSB4XP4	− 7.949885508	5.81E−87	2.77E−83	Down
HLA-DRA	− 6.433931409	2.49E−67	7.92E−64	Down
HLA-DRB1	4.881559101	3.26E−40	3.34E−37	Up
HLA-C	− 3.827439333	3.22E−32	2.36E−29	Down
CTAG2	− 8.620500059	3.63E−32	2.60E−29	Down
TP53TG3B	7.974872805	3.10E−25	1.67E−22	Up
HBG2	3.037410807	4.21E−22	1.92E−19	Up
PREX1	− 3.033488076	4.56E−22	2.04E−19	Down
NT5DC4	4.113416617	6.61E−19	2.52E−16	Up
POP7	− 5.525738397	1.84E−18	6.68E−16	Down
ZNF683	− 2.757126294	1.93E−17	6.75E−15	Down
IFI27	2.467281607	3.95E−15	1.17E−12	Up
FOLH1	5.556989884	8.37E−15	2.33E−12	Up
C4BPA	− 3.345502247	5.40E−14	1.41E−11	Down
TACSTD2	− 2.572481704	6.17E−14	1.59E−11	Down
NXF3	2.86284217	8.33E−14	2.10E−11	Up
HLA-DQB1	2.250981494	1.08E−13	2.70E−11	Up
TBC1D3D	− 3.276789099	3.15E−13	7.58E−11	Down
BOK	2.292169389	4.25E−13	1.01E−10	Up
PRSS50	3.207478048	7.94E−13	1.85E−10	Up
CYP4F3LP	2.286546007	1.02E−12	2.35E−10	Up
TRAC	2.418952461	1.12E−12	2.57E−10	Up
IGHV4-34	− 2.69593089	1.12E−11	2.41E−09	Down
C21orf81	2.196652836	1.34E−11	2.85E−09	Up
BASP1	2.090316943	1.66E−11	3.50E−09	Up

### Expression of m6A regulators and candidate genes correlated with clopidogrel resistance

**Fig. 9:**
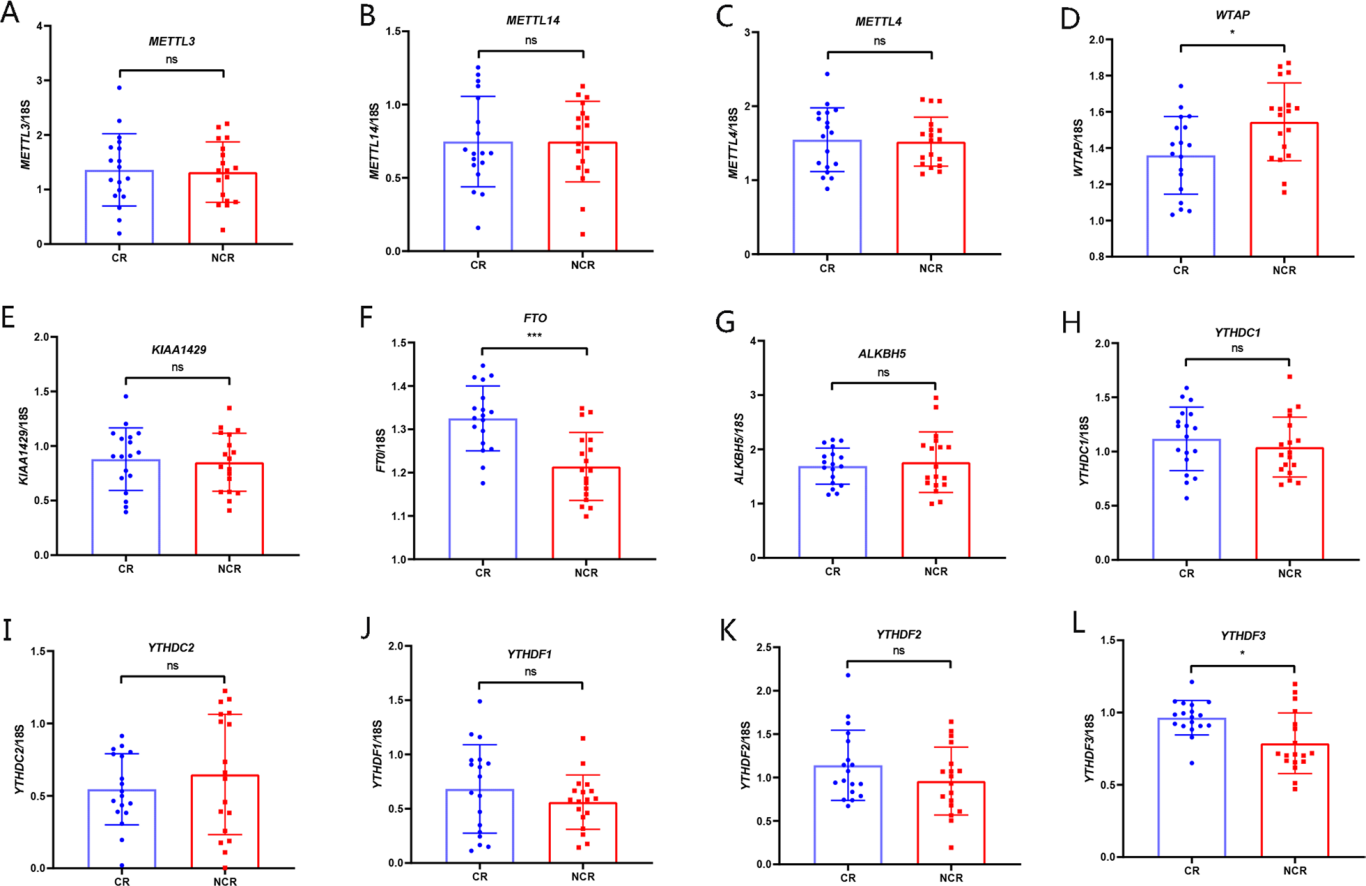
The mRNA expression of m6A regulators in CR and non-CR patients. **A**–**E** m6A writers (METTL3, METTL14, METTL4, WTAP, and KIAA1429), **F**, **G** m6A erasers (FTO and ALKBH5), and **H**–**L** m6A readers (YTHDC1-2 as well as YTHDF1-3)

In an effort to determine whether m6A methylation status was changed in CR patients, with the validated cohort data, we assessed m6A regulator expression in CR and non-CR patients through RT‒PCR. The results indicated that the expression of FTO, a main m6A eraser (*P* = 0.0003), and YTHDF3, a main m6A reader (*P* = 0.0106), was significantly upregulated in CR patients (Fig. 9F and L) compared with non-CR patients (controls). However, the expression of WTAP, a main m6A writer, was lower (*P* = 0.0244) (Fig. 9D). The expression of other m6A regulators was unchanged between the CR and non-CR patient groups (Fig. 9A–C, E, and G–K).

**Fig. 10:**
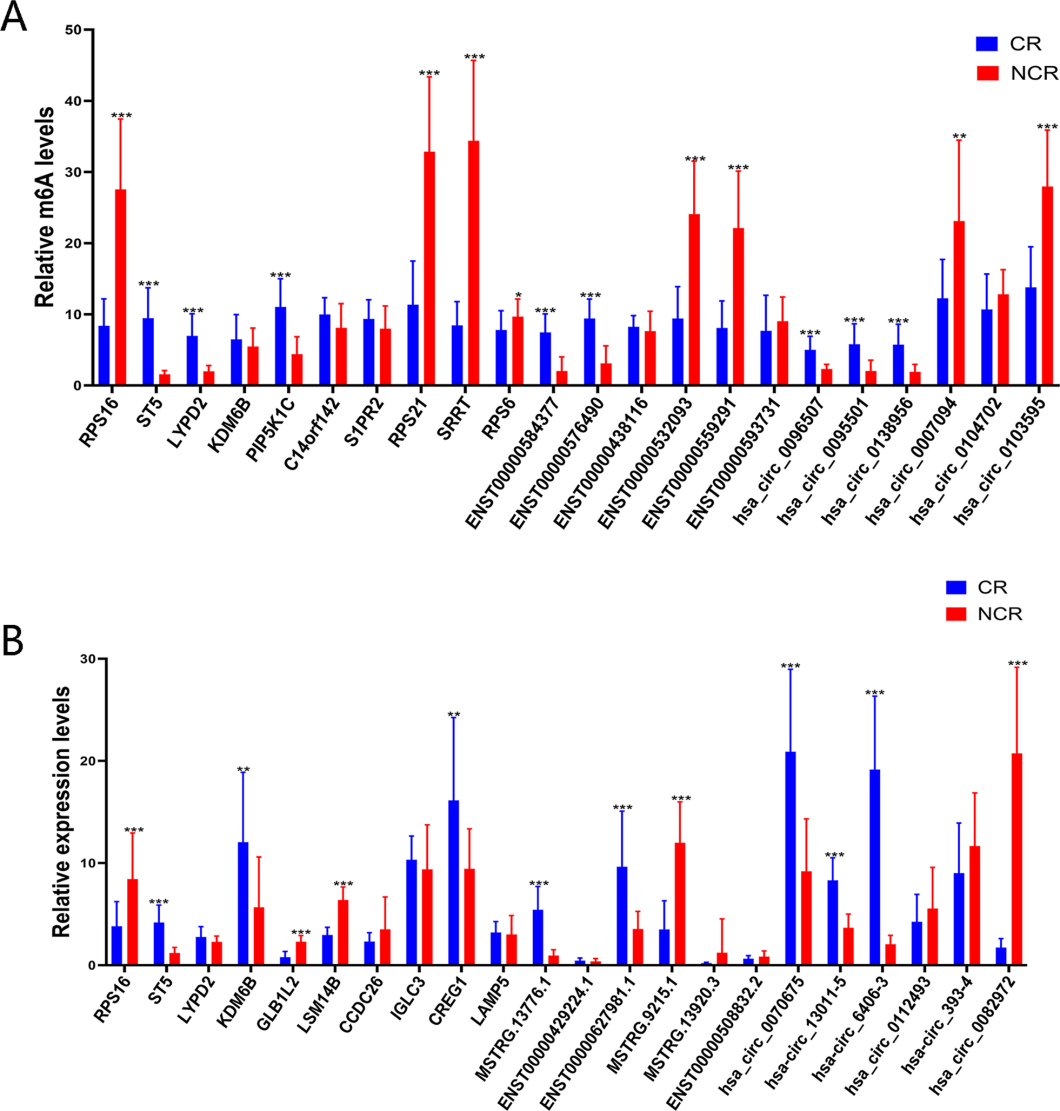
Validation of the m6A-enriched genes and candidate loci. **A** Validation of the m6A-enriched genes as indicated by m6A-immunoprecipitation (IP)-qPCR. **B** Validation of the expression levels of candidate mRNAs, lncRNAs, and circRNAs as determined by RT-qPCR

To validate our m6A-seq results, they were performed gene-specific m6A-IP qPCR assays of four differentially expressed genes identified after a comprehensive analysis (KDM6B, ST5, LYPD2, and RPS16), three hypermethylated mRNAs (PIP5K1C, C14orf142, and S1PR2), and three hypomethylated mRNAs (RPS21, SRRT, and RPS6); three hypermethylated lncRNAs (ENST00000584377 (CSNK1D), ENST00000576490 (RP11-473M20.14), and ENST00000438116 (RPL4P6)) and three hypomethylated lncRNAs (ENST00000532093 (THAP1), ENST00000559291 (C15orf57), and ENST00000593731 (PIK3R2)); and three hypermethylated circRNAs (hsa_circ_0096507 (RSF1), hsa_circ_0095501 (NUCB2), and hsa_circ_0138956 (TMEM2)) and three hypomethylated circRNAs (hsa_circ_0007094 (NDRG3), hsa_circ_0104702 (HOMER2), and hsa_circ_0103595 (TP53BP1)) that might participate in pathological processes in CR patients. We observed almost the same relationships between m6A abundance changes and the expression of these genes (except KDM6B, C14orf142, S1PR2, ENST00000438116, ENST00000593731, and hsa_circ_0104702), confirming the validity of our MeRIP-seq results (Fig. 10A).

Sequentially, to confirm our RNA-seq results, transcript levels of the abovementioned genes (KDM6B, ST5, LYPD2, and RPS16), three upregulated mRNAs (GLB1L2, LSM14B, and CCDC26), lncRNAs (MSTRG.13776.1, ENST00000429224.1, and ENST00000627981.1), and circRNAs (hsa_circ_0070675_CBC1, hsa-circRNA13011-5_CBC1, and hsa-circRNA6406-3_CBC1), as well as downregulated mRNAs (IGLC3, CREG1, and LAMP5), lncRNAs (MSTRG.9215.1, MSTRG.13920.3, and ENST00000508832.2), and circRNAs (hsa_circ_0112493_CBC1, hsa-circRNA393-4_CBC1, and hsa_circ_0082972_CBC1) were also measured in 18 pairs of CR and non-CR patients (Fig. 10B). These findings confirmed that the expression of most of these genes showed similar to the tendency in transcript level changes that we found with the RNA-seq data obtained from the original five pairs of samples.

## Discussion

Antiplatelet treatment is the key treatment for patients with CAD after PCI. Although ticagrelor, a new antiplatelet drug, exhibits rapid, effective, and sustained inhibition of the P2Y12 receptor, patient haemorrhage risk is markedly increased [5]. In clinical practice in China, although ticagrelor reduces cardiovascular mortality and the risk of stent thrombosis in ACS patients, it has an advantage only in patients with low bleeding risk and increases the incidence of cardiovascular events in patients with medium to high bleeding risk [6]. The 2018 COSTIC study proposed that clopidogrel shows a significantly greater clinical benefit for the Chinese population [7]. For patients with stable coronary heart disease or elderly patients, clopidogrel might be more beneficial.

However, clopidogrel resistance significantly increases the risk of adverse cardiovascular and cerebrovascular events. The relevant mechanisms underlying the occurrence of clopidogrel resistance are complex. Various studies have indicated that the extent of clopidogrel resistance is influenced by a series of clinical morbidities, including chronic kidney disease, diabetes mellitus, and smoking and factors such as drug–drug interactions (for instance, proton-pump inhibitors), age, inflammatory status, decreased left ventricular ejection fraction, and increased body mass index [8]. The molecular mechanism underlying clopidogrel resistance has been explored in multiple fields on the basis of sequence differences and epigenetic regulatory factors. First, based on single-nucleotide polymorphisms (SNPs) in drug metabolism-related genes, researchers found that ABCB1 genes are involved in duodenal transport; CYP family genes are involved in liver metabolism; PON-1 genes and P2Y12 genes are related to platelet activation. Preliminary research by this project team revealed that the ABCB1 C3435T polymorphism is associated with platelet activity and MACEs in ACS patients taking clopidogrel [9]. Another genome-wide association study suggested that genetic variations in genes such as CYP2C19, PEAR1, and N6AMT1 are associated with different antiplatelet effects and clinical endpoint events related to clopidogrel [10]. Second, the up- or downregulation of noncoding RNA also affects the antiplatelet activity of clopidogrel. A recent omics study suggested that upregulation of the lncRNAs NONHSAT083775.2 and NONHSAT107804.2 or downregulation of the lncRNA NONHSAT133455.2 led to clopidogrel resistance [11]. Another study with DM animal models revealed that lncRNA MT1P3 (metallothionein 1 pseudogene 3) promoted the expression of P2Y12 receptors by sponging miR-126, thereby increasing platelet activity [12]. Moreover, miRNAs participate in the regulation of clopidogrel resistance [13]; for instance, miRNA-26a upregulation increased the clopidogrel resistance risk in patients undergoing PCI [14], and platelet-derived miRNAs (such as miRNA-24-3p, miRNA-142-3p, and miRNA-411-3p) might be potential biomarkers. In addition, the advancement of epigenetic studies has provided a new perspective on the clopidogrel resistance mechanism. Our group found that in CAD patients with hyperlipidaemia, CpG4 methylation of the PON1 promoter resulted in low mRNA expression of PON1, leading to clopidogrel resistance [15]. Our latest research was based on a ChIP analysis of methylated DNA from clopidogrel resistance samples and revealed the identification of 979 hypermethylation sites and 6119 hypomethylation sites, and four sites (cg23371584, cg15971518, cg04481923, and cg22507406) were verified to be clopidogrel resistance diagnostic markers [16]. In addition, N6-methyladenosine (m6A) might play a vital role in posttranscriptional modification.

In the present study, by preparing the RNA transcriptome expression profiles and validating the findings with samples from a larger cohort, we discovered that the expression of mRNAs (ST5, KDM6B, GLB1L2, and LSM14B), lncRNAs (MSTRG.13776.1 and ENST00000627981.1), and circRNAs (hsa_circ_0070675_CBC1, hsa-circRNA13011-5_CBC1, and hsa-circRNA6406-3_CBC1) was upregulated in CR patients, while mRNAs (RPS16 and CREG1), lncRNAs (MSTRG.9215.1), and circRNAs (hsa_circ_0082972_CBC1) were downregulated in CR patients. This is the first study to report on the landscape of the transcriptome based on sequencing of samples from patients with clopidogrel resistance, and it revealed some CR-related biomarkers. Although the results need to be validated with data from a larger sample and from a broader population, the markers it revealed will provide a basis for early detection in clinical practice. Thus, the ST5, KDM6B, and CREG1 genes deserve our continued attention. First, the *Cryptococcus neoformans* sequence type 5 (ST5) lineage can be used to infect immunocompetent hosts and increase the blood platelet count [17]. In our study, the ST5 level was higher in CR patients, which might have been due to a high level of ST5 increasing the platelet count, leading to clopidogrel resistance. Second, lysine-specific demethylase 6B (KDM6b) is involved in vascular remodelling, and it can promote neointima formation by inhibiting the activation of Nox4 signalling in autophagy [18]. Third, the cellular repressor of E1A-stimulated genes (CREG1), which was downregulated in CR patients, promotes HUVEC proliferation and prevents endothelial cells (ECs) from undergoing apoptosis. Through ILK-Cdc42 activation, CREG1 can also increase EC filopodia formation and vascular assembly to promote neovascularization [19]. Likewise, a latest study uncovered the role of CREG1 in the regulation of megakaryocyte maturation and thrombopoiesis [20]. Hence, ST5, KDM6B, and CREG1 might be therapeutic targets for clopidogrel resistance and vascular diseases. Future researches might further validate the findings of this article.

Moreover, we carried out MeRIP-seq with samples from patients with low clopidogrel responses and found that m6A modification of mRNA on ST5, LYPD2, RPS16, PIP5K1C, RPS21, SRRT, and RPS6 affected mRNA expression, resulting in clopidogrel resistance. In addition, the m6A regulators FTO, YTHDF3, and WTAP were also differentially expressed in CR and non-CR patients. These findings may indicate that upregulated FTO or downregulated WTAP causes reduced expression of related genes. FTO, a key m6A enzyme, is involved in the pathophysiological processes leading to myocardial hypertrophy and cardiac dysfunction [21]. Depending on the m6A modification, FTO regulates the cardiac function after systolic heart dysfunction in model rats and influences cardiac remodelling and repair [22]. Loss of the m6A reader YTHDF2 promotes demethylation of H3K27me3, leading to enhanced production of proinflammatory cytokines [23]. Moreover, WTAP has been reported to regulate the progression of myocardial ischaemia/reperfusion injury via the YTHDF1/FOXO3a signalling pathway [24]. After our comprehensive analysis, we speculated that the higher degree of m6A methylation on ST5 mRNA might lower its expression and result in clopidogrel resistance. The intrinsic molecular mechanism needs to be confirmed with cell experiments.

Furthermore, in this study, we proposed that inflammatory pathways (such as the NF-κB, Wnt, and Notch signalling pathways) as well as the insulin signalling pathway participate in the regulation of clopidogrel resistance. For example, through the NF-κB signalling pathway, nucleotide-binding oligomerization domain 2 (NOD2) mediates P2Y12 upregulation and increases platelet activation, resulting in clopidogrel resistance [25]. On the other hand, abnormal excretion of insulin leads to an increase in platelet activity, and hyperglycaemia activates the protein kinase C pathway by saccharifying proteins on the surface of platelets, inducing the expression of P selectin, reducing the fluidity of the cell membrane, and thus increasing the platelet adhesion rate and enhancing platelet activity [26]. FTO is an important factor in blood glucose regulation, because it regulates insulin signalling-related gene expression possibly through m6A modification. Research suggested that FTO is associated with insulin secretion and abnormal blood glucose levels, and it may interact with transcription factors (STAT3, CREB, ATF4, and FoxO1) and thus change their activity, regulating the expression of liver gluconeogenesis genes (PCK1 and G6PC) [27]. A functional experiment with pancreatic ß cells showed that increasing the expression of FTO led to an increase in the gluconeogenesis rate and that reducing the activity of FTO led to the opposite results [28]. Hence, FTO may regulate insulin signalling-related gene expression through m6A modification, changing the antiplatelet effect of clopidogrel. Further animal and cell experiments are needed to confirm this possibility.

To the best of our knowledge, our research is the first to report RNA transcriptome expression profiles and potential impact of m6A methylation on clopidogrel resistance. Although we made great efforts, this research still has unavoidable limitations. First, it was a single-centre study, and the studied population was not large. We will further expand the sample size to verify our conclusions. Second, PBMCs are collected from peripheral blood, and their status may be influenced by various factors, such as individual differences, etc., which may interfere with the interpretation of RNA transcriptome, and RNA methylation may be influenced by other factors, such as environment factors and coexisting diseases. These influences may have affected our results. Finally, knockout animal models need to be used for in vivo validation studies, and an in-depth mechanistic investigation will increase our understanding in the future.

## Conclusions

In general, our present study revealed the RNA transcriptome expression profiles in CR and non-CR patients and showed that the expression of some mRNAs (ST5, KDM6B, GLB1L2, and LSM14B), lncRNAs (MSTRG.13776.1 and ENST00000627981.1), and circRNAs (hsa_circ_0070675_CBC1, hsa-circRNA13011-5_CBC1, and hsa-circRNA6406-3_CBC1) was upregulated in CR patients, while that of other mRNAs (RPS16 and CREG1), lncRNAs (MSTRG.9215.1), and circRNAs (hsa_circ_0082972_CBC1) was downregulated in CR patients. Moreover, m6A regulators (FTO, YTHDF3, and WTAP) were differentially expressed. By MeRIP-seq, we found that 2919 hypermethylated and 2519 hypomethylated mRNAs, 192 hypermethylated and 391 hypomethylated lncRNAs, and 375 hypermethylated and 546 hypomethylated circRNAs were markedly changed in CR patient samples. The joint analysis of mRNA m6A peaks and expression revealed that the mRNAs (such as ST5, LYPD2, and RPS16) might cause clopidogrel resistance because their expression is changed via m6A modification. These are critical regulators that interfere with the epigenetic regulation of clopidogrel resistance via the inflammatory pathway and insulin signalling pathway, and these findings provide new insights useful for CR mechanistic research and will help clinicians identify individualized treatments more effectively.

## Materials and methods

### Patients

Between April and December 2020, a total of 23 CR and 23 non-CR controls in the First Affiliated Hospital of Ningbo University were enrolled in the present research. All the enrolled patients were diagnosed with ACS and were treated of PCI with drug-eluting stents to re-establish the coronary artery blood supply. The case group conformed to the standard for a CR diagnosis, which is reaction units of P2Y12 (PRU) greater than 240 [29] as measured by the VerifyNow P2Y12 assay (Accumetrics, Inc., San Diego, California). The enrolled CR and non-CR patients did not have a history of acute or severe infection, disordered kidney or hepatic function, active haemorrhage, or rheumatoid-related diseases.

The clinical data, demographic data, and laboratory indices of these patients were collected. These data included BMI, age, sex, drinking rate, smoking rate, comorbidities (diabetes, hypertension, or hyperlipidaemia), medications, aTnI, ALT, AST, LDL-C, creatinine, uric acid, platelets, pro-BNP status, and other measures.

This research was approved by the Ethics Committee of the First Affiliated Hospital of Ningbo University, and every individual signed a written informed consent form before the study was initiated. The study was performed in accordance with Declaration of Helsinki principles.

### RNA preparation

Peripheral blood was collected from 23 CR patients and an equal number of non-CR subjects after clopidogrel therapy that was administered longer than 6 months. Samples from 10 of these patients were used for a comprehensive analysis of RNA m6A modification (the five pairs of CR and non-CR patients were well matched), while samples from the remainder of the patients (18 CR and 18 non-CR patients) were used to validate the results.

Peripheral samples were obtained from the patients described above and placed into an ETDA anticoagulant vacutainer. Peripheral blood mononuclear cells (PBMCs) were isolated as described previously [30]. Total RNA was obtained with TRIzol reagent (Invitrogen, USA), and the RNA purity and concentration were determined with a NanoDrop 2000 spectrophotometer (Thermo Fisher Scientific, USA).

### RT-PCR

RT-PCR was performed with cDNA. First, reverse transcription was performed with RNA by SuperScript III Reverse Transcriptase (Invitrogen, USA) according to the manufacturer’s instructions. The following reagents were added in sequence to a 0.5-ml PCR tube: 2 µl of first-strand cDNA; 2 µl of upstream primer (10 pM); 2 µl of downstream primer (10 pM); 4 µl of dNTP (2 mM); 5 µl of 10 × PCR buffer; and 1 µl of Taq enzyme (2 u/ul). Then, PCR was performed according to the following programme: 95 °C, 10 min; 30 PCR cycles (95 °C, 10 s and 60 °C, 60 s), and amplification reaction 28–32 cycles (95 °C, 10 s; 60 °C, 60 s; and 95 °C, 15 s) and slowly warmed from 60 to 99 °C to establish the melting curve. The housekeeping genes U6/GAPDH were utilized as the internal controls to ensure the reliability and accuracy of the experimental results.

### RNA-seq and MeRIP-seq

RNA-seq and MeRIP-seq were performed at CloudSeq Biotech Inc. according to a procedure described previously [31]. Briefly, after RNA isolation, the total RNA was fragmented into approximate 100 nt sequences, and those fragments were incubated with anti-m6A antibody (Manga) for 2 h at 4 °C. Then, total RNA was incubated with prepared beads for 2 h at 4 °C. Finally, the complexes were washed from the beads and purified with TE buffer. Both the RNA that was not immunoprecipitated and IP samples were used for library construction. The library quality was evaluated with a Bioanalyzer 2100 system. The library was sequenced on an Illumina HiSeq instrument. All the sequence data have been uploaded Supplement date (Additional file 4: Stable 4, Additional file 5: Stable 5, and Additional file 6: Stable 6).

### Data analysis of MeRIP-seq and RNA-seq

Paired-end reads were obtained using a Illumina HiSeq 4000 sequencer, and the quality was controlled on the basis of a Q30 sore. Then, 3′ adaptor trimming and low-quality reads were removed by Cutadapt software (version: 1.9.3). Then, all the clean reads in the library were aligned to the reference genome (UCSC HG19) by Hisat2 software (version: 2.0.4). DCC software was used to recognize circRNAs based on the results of STAR alignment. Methylated sites on RNAs (m6A peaks) were recognized using MACs software. The recognized methylated sites were used for motif enrichment analysis by HOMER [32]. In addition, the R package MetaPlotR was used to characterize the m6A distribution. diffReps was used to identify differentially methylated sites at threshold levels of |log_2_ FC| higher than 1 and a *P* value less than 0.05. Scripts were written in-house to identify and select the differentially methylated sites that overlapped with mRNA exons. For RNA-seq, raw counts were obtained with HTSeq software (version 0.8.2), followed by normalization using edge R software. Genes of interest were directly visualized with Integrative Genomics Viewer (IGV; version: 2.3.68).

### GO and KEGG pathway enrichment analysis

Gene Ontology (GO) enrichment analysis in three categories: biological process (BP), cellular component (CC), and molecular function (MF) categories, was performed to identify the function of identified genes related to differential peaks or differential expression of mRNAs, lncRNAs, and circRNAs (http://www.geneontology.org). GO analysis of differentially expressed peaks or RNAs was performed by using R software based on hypergeometric distribution. The number of genes related to altered peaks or enriched in each GO term was counted, and the related gene attributes annotated with a GO term were identified (http://www.geneontology.org).

In addition, Kyoto Encyclopedia of Genes and Genomes (KEGG) pathway analysis (www.genome.jp/kegg) was performed by using R software, and a hypergeometric distribution test was performed to evaluate the significance of genes related to altered peaks or that of differential expression of mRNAs, lncRNAs, and circRNAs in association with each pathway term.

### m6A-IP-qPCR and RT-qPCR

Twenty-two genes with differentially methylated sites as determined according to MeRIP-seq were verified by RT-qPCR. A small amount of fragmented RNA was used as the input control. The remainder of the RNA was incubated with anti-m6A antibody-coupled beads. The m6A-interacting RNAs were then immunoprecipitated and eluted from the beads. Both input control and m6A-IP samples were subjected to RT-qPCR with gene-specific primers.

### Statistical analysis

All statistical analyses were performed using GraphPad Prism 8.0 software. The classification data were described as percentages, and the differences between individual groups were determined by Chi-square test. The measurement data were expressed as the mean ± standard deviation, and the significance of differences between two groups was determined by t-test or Pearson correlation test. A two-tailed *P* value less than 0.05 was considered to be statistically significant.

### Supplementary Information

**Additional file 1: STable 1.** Differential mRNA expression in CR and non-CR patients.

**Additional file 2: STable 2.** Differential lncRNA expression in CR and non-CR patients.

**Additional file 3: STable 3.** Differential circRNA expression in CR and non-CR patients.

**Additional file 4: STable 4.** Methylated RNA sites in mRNA.

**Additional file 5: STable 5.** Methylated RNA sites in lncRNA.

**Additional file 6: STable 6.** Methylated RNA sites in circRNA.

## Data Availability

The datasets generated and/or analysed during the current study are not publicly available due to the latest Requirements for Genomic Data Management in China, but are available from the corresponding author on reasonable request.

## References

[1] Udell JA, Bonaca MP, Collet JP, Lincoff AM, Kereiakes DJ, Costa F, Lee CW, Mauri L, Valgimigli M, Park SJ (2016). Long-term dual antiplatelet therapy for secondary prevention of cardiovascular events in the subgroup of patients with previous myocardial infarction: a collaborative meta-analysis of randomized trials. Eur Heart J.

[2] Pahl MC, Grant SFA, Leibel RL, Stratigopoulos G (2023). Technologies, strategies, and cautions when deconvoluting genome-wide association signals: FTO in focus. Obes Rev.

[3] Yang Y, Shen F, Huang W, Qin S, Huang JT, Sergi C, Yuan BF, Liu SM (2019). Glucose is involved in the dynamic regulation of m6A in patients with type 2 diabetes. J Clin Endocrinol Metab.

[4] Mathiyalagan P, Adamiak M, Mayourian J, Sassi Y, Liang Y, Agarwal N, Jha D, Zhang S, Kohlbrenner E, Chepurko E (2019). FTO-dependent N(6)-methyladenosine regulates cardiac function during remodeling and repair. Circulation.

[5] Wiviott SD, Steg PG (2015). Clinical evidence for oral antiplatelet therapy in acute coronary syndromes. Lancet.

[6] Wang HY, Li Y, Xu XM, Li J, Han YL (2018). Impact of baseline bleeding risk on efficacy and safety of ticagrelor versus clopidogrel in Chinese patients with acute coronary syndrome undergoing percutaneous coronary intervention. Chin Med J.

[7] Sun Y, Li C, Zhang L, Yu T, Ye H, Yu B, Tao M, Jiang J, Yan J, Wang Y (2019). Clinical outcomes after ticagrelor and clopidogrel in Chinese post-stented patients. Atherosclerosis.

[8] Alkattan A, Alkhalifah A, Alsalameen E, Alghanim F, Radwan N (2022). Polymorphisms of genes related to phase II metabolism and resistance to clopidogrel. Pharmacogenomics.

[9] Su J, Xu J, Li X, Zhang H, Hu J, Fang R, Chen X (2012). ABCB1 C3435T polymorphism and response to clopidogrel treatment in coronary artery disease (CAD) patients: a meta-analysis. PLoS ONE.

[10] Zhong WP, Wu H, Chen JY, Li XX, Lin HM, Zhang B, Zhang ZW, Ma DL, Sun S, Li HP (2017). Genomewide association study identifies novel genetic loci that modify antiplatelet effects and pharmacokinetics of clopidogrel. Clin Pharmacol Ther.

[11] Xie W, Huang B, Yin Q, Chen S (2019). Differential expression of lncRNA in patients with coronary artery disease plus clopidogrel resistance. J Central South Univ Med Sci.

[12] Zhou M, Gao M, Luo Y, Gui R, Ji H (2019). Long non-coding RNA metallothionein 1 pseudogene 3 promotes p2y12 expression by sponging miR-126 to activate platelet in diabetic animal model. Platelets.

[13] Li X, Yao Q, Cui H, Yang J, Wu N, Liu Y, Zhou Y, Zhang Y, Su J, Xia Y (2021). MiR-223 or miR-126 predicts resistance to dual antiplatelet therapy in patients with ST-elevation myocardial infarction. J Int Med Res.

[14] Giantini A, Timan IS, Dharma R, Sukmawan R, Setiabudy R, Alwi I, Harahap AR, Listiyaningsih E, Partakusuma LG, Tansir AR (2022). The role of clopidogrel resistance-related genetic and epigenetic factors in major adverse cardiovascular events among patients with acute coronary syndrome after percutaneous coronary intervention. Front Cardiovasc Med.

[15] Su J, Li J, Yu Q, Xu X, Wang J, Yang J, Li X, Chen X (2019). Association of PON1 gene promoter DNA methylation with the risk of Clopidogrel resistance in patients with coronary artery disease. J Clin Lab Anal.

[16] Yang J, Yu Q, Xu Z, Zheng N, Zhong J, Li J, Liu Y, Xu H, Su J, Ji L (2020). Clopidogrel resistance is associated with DNA methylation of genes from whole blood of humans. Front Genet.

[17] Tian Y, Wang J, Shen Y, Zhao J, Hu J, Zhu X, Zhu M, Guan M (2023). Characteristics and prognostic risk factors of patients with sequence type 5 lineage-associated cryptococcosis in China. Int J Infect Dis.

[18] Luo X, Yang D, Wu W, Long F, Xiao C, Qin M, Law BY, Suguro R, Xu X, Qu L (2018). Critical role of histone demethylase Jumonji domain-containing protein 3 in the regulation of neointima formation following vascular injury. Cardiovasc Res.

[19] Yan C, Fang P, Zhang H, Tao J, Tian X, Li Y, Zhang J, Sun M, Li S, Wang H (2014). CREG1 promotes angiogenesis and neovascularization. Front Biosci (Landmark Ed).

[20] Song H, Li J, Peng C, Liu D, Mei Z, Yang Z, Tian X, Zhang X, Jing Q, Yan C (2023). The role of CREG1 in megakaryocyte maturation and thrombocytopoiesis. Int J Biol Sci.

[21] Mauer J, Luo X, Blanjoie A, Jiao X, Grozhik AV, Patil DP, Linder B, Pickering BF, Vasseur JJ, Chen Q (2017). Reversible methylation of m(6)Am in the 5′ cap controls mRNA stability. Nature.

[22] Wang Y, Wang Y, Gu J, Su T, Gu X, Feng Y (2022). The role of RNA m6A methylation in lipid metabolism. Front Endocrinol.

[23] Wu C, Chen W, He J, Jin S, Liu Y, Yi Y, Gao Z, Yang J, Yang J, Cui J (2020). Interplay of m(6)A and H3K27 trimethylation restrains inflammation during bacterial infection. Sci Adv.

[24] Wang H, Fu L, Li Y, Wei L, Gu X, Li H, Li J, Wen S. m6A methyltransferase WTAP regulates myocardial ischemia reperfusion injury through YTHDF1/FOXO3a signaling. Apoptosis 2023. 10.1007/s10495-023-01818-4 36894806

[25] Zhong H, Waresi M, Zhang W, Han L, Zhao Y, Chen Y, Zhou P, Chang L, Pan G, Wu B (2021). NOD2-mediated P2Y(12) upregulation increases platelet activation and thrombosis in sepsis. Biochem Pharmacol.

[26] Ferreiro JL, Angiolillo DJ (2011). Diabetes and antiplatelet therapy in acute coronary syndrome. Circulation.

[27] Dayeh T, Volkov P, Salo S, Hall E, Nilsson E, Olsson AH, Kirkpatrick CL, Wollheim CB, Eliasson L, Ronn T (2014). Genome-wide DNA methylation analysis of human pancreatic islets from type 2 diabetic and non-diabetic donors identifies candidate genes that influence insulin secretion. PLoS Genet.

[28] Mizuno TM (2018). Fat mass and obesity associated (FTO) gene and hepatic glucose and lipid metabolism. Nutrients.

[29] Marcucci R, Gori AM, Paniccia R, Giusti B, Valente S, Giglioli C, Buonamici P, Antoniucci D, Abbate R, Gensini GF (2009). Cardiovascular death and nonfatal myocardial infarction in acute coronary syndrome patients receiving coronary stenting are predicted by residual platelet reactivity to ADP detected by a point-of-care assay: a 12-month follow-up. Circulation.

[30] Gautam A, Donohue D, Hoke A, Miller SA, Srinivasan S, Sowe B, Detwiler L, Lynch J, Levangie M, Hammamieh R (2019). Investigating gene expression profiles of whole blood and peripheral blood mononuclear cells using multiple collection and processing methods. PLoS ONE.

[31] Meyer KD, Saletore Y, Zumbo P, Elemento O, Mason CE, Jaffrey SR (2012). Comprehensive analysis of mRNA methylation reveals enrichment in 3′ UTRs and near stop codons. Cell.

[32] Heinz S, Benner C, Spann N, Bertolino E, Lin YC, Laslo P, Cheng JX, Murre C, Singh H, Glass CK (2010). Simple combinations of lineage-determining transcription factors prime cis-regulatory elements required for macrophage and B cell identities. Mol Cell.

